# Career influences among final year dental students who plan to enter private practice

**DOI:** 10.1186/1472-6831-14-18

**Published:** 2014-03-08

**Authors:** Benjamin M Nashleanas, Susan C McKernan, Raymond A Kuthy, Fang Qian

**Affiliations:** 1 College of Dentistry, University of Iowa, Iowa City, IA, USA; 2 Department of Preventive and Community Dentistry, College of Dentistry, University of Iowa, Iowa City, IA, USA; 3 Public Policy Center, University of Iowa, Iowa City, IA, USA

## Abstract

**Background:**

Existing research about the influence of educational debt on students’ decision to enter general practice immediately after graduation is conflicting. Other potential factors that could affect this decision include the influence of a spouse or other family member, the importance of a mentoring dentist, and how students perceive the burden of their debt. The goal of this study was to examine the importance of debt on career decision-making while also considering the role of other influences.

**Methods:**

Responses to a self-completed questionnaire of all final (fourth) year students at the University of Iowa College of Dentistry from 2007 through 2010 were analyzed to identify the importance of educational debt and the influence of spouses, other family members, and mentoring dentists in the decision to enter private general practice immediately after graduation. Statistical analysis included bivariate tests (t-tests and Chi-square tests) and multivariable logistic regression.

**Results:**

58.9% of respondents (N = 156) planned to immediately enter private practice after dental school. Bivariate analyses revealed women to be more likely to enter private practice than their male counterparts (69.0% vs. 51.8%, p = .006). Students planning to enter practice immediately did not differ significantly from those with other career plans on the basis of marital status or having a family member in dentistry. Anticipated educational debt of at least $100,000 was positively associated with plans to enter private practice immediately after graduation. Self-reported importance of educational debt was not associated with career plans. However, the influence of a spouse, other family members, and family dentists were also positively associated with the decision to enter private practice. These factors all maintained significance in the final multivariable model (p < 0.05); however, educational debt of at least $100,000 was the strongest predictor of plans to enter private practice (OR = 2.34; p = 0.023).

**Conclusions:**

Since the 1970s, increasing numbers of dentists in the U.S. have pursued specialty training after dental school. However, rising educational debts may counter this trend as increasing numbers of dentists choose to immediately pursue general dentistry at graduation. This project has demonstrated the significant influence of educational debt, beyond other external influences.

## Background

The majority of American dentists are engaged in general practice. However, since the 1970s, the proportion of general dentists has steadily declined. In 1970, specialists made up less than 10% of all dentists in the U.S. Now, approximately 22% of active dentists are specialists and this number is expected to increase over the next decade [1]. Currently, half of new graduates enter private general practice immediately after dental school [2]. As the proportion of general dentists has declined, so has the proportion entering academics and research, while the proportion of specialists has increased [3].

With increasing national emphasis on preventive services provided in primary care settings, ensuring a strong general dentist workforce is of paramount importance for U.S. dental schools. As the Affordable Care Act (ACA) is implemented over the next few years, over 150,000 uninsured Iowans are expected to gain insurance coverage, including dental benefits, through the Iowa Health and Wellness Plan [4]. A sudden influx of newly insured adults with a backlog of untreated dental needs will turn to general dentists in private practice and public health settings for care.

Concurrent with the changing profile of the dentist workforce, several other trends of interest have received considerable attention. Within the profession, the costs of education continue to rapidly escalate. Since 1990, average educational debt at graduation from public dental school has increased approximately three-fold in constant dollars [2]. Average educational debt among graduates from public dental schools is approximately $158,000. Thirty-percent of new graduates owe over $250,000 [2].

Several recent studies have demonstrated that increased educational debt is associated with a decreased intention to pursue specialty training after dental school [5-7]. Dhima et al. found that students with the highest levels of expected debt were more likely to plan to enter general dentistry or advanced training in general dentistry immediately after dental school. Among Canadian dental students, 77% of those surveyed were considering a career in general dentistry; however, if educational debt were not a factor, only 12% reported that they would be considering general dentistry [6].

The burden of debt has workforce implications beyond the decision whether or not to specialize. Analysis of data from the 2007 American Dental Education Association (ADEA) survey of dental school seniors found that students with high levels of debt were the least likely to consider a career in public service [8]. This contrasts with analysis of more recent ADEA data (2011) that did not find a correlation between debt and the intention to practice in underserved areas [9].

Among U.S. medical students, the role of educational debt on career choice has been researched with mixed results. One study found that students with higher educational debt were less likely to enter primary care [10]. More recent findings have demonstrated that educational debt is not independently associated with employment in academia [11,12]. However, educational debt appears to affect male and female health care providers differently. Female physicians with higher educational debt were more likely to hold a faculty position than their male counterparts [11].

Despite being more likely than men to choose academic careers in dentistry [13,14], women have not progressed into top leadership positions proportionally [15]. This phenomenon is also seen in medicine, where admission into medical school has been approximately equal among men and women since 2003, but large disparities in faculty gender exist [16]. Only 17% of tenured faculty and 7% of medical school deans are women.

Additionally, dentistry has seen another shift as increasing numbers of women enter the traditionally male-dominated workforce. From 1985 through 2005, the number of female dentists increased from 25 to 45% of new graduates [17]. The American Dental Association (ADA) estimates that 21% of all professionally active dentists in the U.S. are women [18]. Given the rising rates of new females entering the profession and the aging of the current workforce, women will continue to increase their presence in dentistry over the next several decades.

External trends with potential influence on America’s dentist workforce are the increasing number of dual income households and shifting general roles among younger professionals. Approximately half of male dentists and one-quarter of female dentists feel that their partners had made career adjustments to accommodate their dental careers [19]. This discrepancy highlights the value that men and women ascribe to their spouses’ career opportunities: a survey of prospective pediatric dentistry residents found that females placed more importance on their spouses’ careers than male residents [20].

Male and female dentists may also prioritize the influence of other individuals differently. Female dental students pursuing advanced education degrees have rated proximity to family as being significantly more important than male students [21].

A recent task force convened by the ADEA reported that the current evidence describing the relationship between educational debt and career plans after graduation is inconclusive [22]. The general assumption seems to be that students with the highest levels of debt (and the greatest concern about the burden of debt) will choose career options that will allow them to quickly pay off that debt. However, the income differential between general dentists and specialists may entice students to forego immediate earnings and opt to specialize. In 2009, the average net income for independent dental specialists was $306,000, compared to $193,000 for general dentists [23].

Given the mixed findings regarding the influence of debt and the evidence for other external influences, this current study was designed to study the relative importance of educational debt on post-graduation career plans of fourth year dental students, while accounting for the influence of important people in students’ lives. The influence of spouses, other family members, faculty mentors, and family dentists are all hypothesized to affect students’ career plans and potentially modify the influence of debt.

## Methods

This study is part of a longitudinal series of surveys administered to the predoctoral classes of 2007 through 2010 at the University of Iowa College of Dentistry. The original 24-item survey collected background data from all four classes and administered with verbal instructions during the first week of dental school in August 2006. Thereafter, a nearly identical survey was given at the start of the academic year for each of the three following years. This project as designed was exempt from human subjects research oversight by the University of Iowa Institutional Review Board.

Participation was voluntary and anonymous. Individual responses were linked across years through the use of a self-generated code. The questionnaire was pre-tested for face validity by collegiate faculty, residents, and dental students. Along with demographic questions, students were asked to provide information about their home town, previous work experience, and career intentions after graduation from dental school.

Students were asked to rate the influence of various individuals on their post-graduation career plans through a series of 10-point Likert-like items (1 = No influence, 10 = Very much). Three items examined the influence of mentoring dentists including a family dentist, dental specialist, or faculty member. Other sources of potential influence included spouse, spouse’s occupation, family other than spouse, and parents’ occupation. Missing values were recoded as level 1, indicating that this factor did not influence their career plans at all.

The importance of educational debt was assessed with two survey items. One question asked students to estimate their anticipated debt at graduation and a second item asked students to rate the influence of educational debt on their career plans. Because the question about anticipated debt used different response options across the study period and demonstrated a bimodal frequency distribution, this variable was dichotomized as less than $100,000 or at least $100,000 for analysis. Previous studies have also examined the influence of debt using these categories [24].

The primary outcome of interest was reported career plan immediately after dental school, dichotomized as private practice versus other plans. Other career options included: dental school faculty; military; specialty or other training; other dental occupation; other non-dental occupation; and unknown.

Demographic characteristics were summarized and bivariate comparisons between predictors and post-graduation career plans were assessed using Chi-square tests for categorical variables and Mann-Whitney U tests for continuous variables. Multivariable logistic regression analysis was used to examine the influences of factors on plans to enter practice immediately after graduation while controlling for demographic characteristics. Survey items that demonstrated statistically significant bivariate relationships with career plans were included in the regression model. Two additional variables were also retained in the regression model due to their conceptual relevance. Marital status was retained because of its association with influence of a spouse’s occupation and having a family member in dentistry was retained because of its relationship with the influence of family other than a spouse.

Conceptually plausible interaction terms were considered, including interactions between marital status and influence of spouse’s job, gender and marital status, along with gender and influence of spouse’s job. Models were compared using log likelihood statistics. Multicollinearity was assessed by examining condition indices and eigenvalues. Condition indices over 30 were considered to be suggestive of multicollinearity. Statistical analyses were conducted using IBM SPSS Statistics (version 20). A significance level of 0.05 was used for all hypothesis testing.

## Results

The overall response rate among final year dental students was 90.1%, with 265 of 294 students responding. The ratio of male to female respondents (55% and 45%, respectively) reflected the ratio of enrolled dental students (57% and 43%). Table 1 shows the demographic characteristics and career plans of the sample population. Approximately 59% of respondents indicated that they planned to enter practice immediately after dental school; 28% of students planned to pursue specialty training after graduation. Half of the dental students anticipated at least $150,000 in educational debt at graduation, while fewer than 5% of dental students anticipated having no educational debt (Table 1).

**Table 1:** Dental student characteristics (n = 265)

	**N (%)**
**Gender**	
Male	139 (55.2%)
Female	113 (44.8%)
**Graduation Year**	
2007	66 (24.9%)
2008	58 (21.0%)
2009	69 (26.0%)
2010	72 (27.2%)
**Marital Status**	
Married	114 (43.0%)
Other (single or divorced)	151 (57.0%)
**Plans immediately after school**	
Private practice	156 (58.9%)
Military	17 (6.4%)
Specialty/other training	74 (27.9%)
Other dental occupation	5 (1.9%)
Unknown	13 (4.9%)
**Anticipated educational debt**	
No debt	13 (4.9%)
$1-49,999	19 (7.2%)
$50,000-99,999	18 (6.8%)
$100,000-149,000	83 (31.3%)
$150,000+	132 (49.8%)

**Figure 1:**
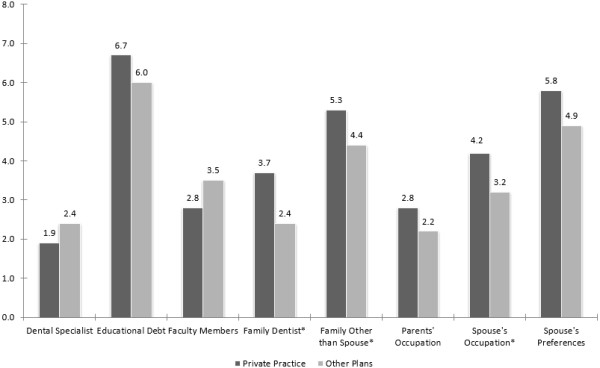
**Self-reported influence of factors† among dental students planning to enter private practice or with other career plans.** †Influence rated from 1 (no influence) to 10 (very much). *Significant at p < 0.05.

There were no statistically significant differences between graduating classes with regards to the proportion of students planning to enter practice immediately after graduation (Table 2). However, students who planned to enter practice immediately after graduation were significantly more likely to be female and to anticipate educational debt greater than $100,000 (Table 2).Mean levels of influence from spouses, other family members, faculty mentors, and family dentists ranged from 2.5 (parent’s occupation) to 5.5 (spouse’s preferences) (Figure 1). When asked to rate the influence of educational debt on post-graduation career plans (1 = Not at all, 10 = Very much), mean response level was 6.4 (SD 3.0). Forty-six percent of dental students rated the influence of educational debt as an 8 or higher. Despite having the highest overall mean level of influence (Figure 1), the influence of educational debt did not differ significantly between students planning to enter private practice and those with other career plans.

**Table 2:** Associations between career plans and dental student characteristics

	**Private practice**	**Other plans†**	**Sig.**
	**N = 156**	**N = 109**	
	**N (%)**	**N (%)**	
**Graduating class**			0.886
2007	39 (59.1%)	27 (40.8%)	
2008	33 (56.9%)	25 (43.1%)	
2009	39 (56.5%)	30 (43.5%)	
2010	45 (62.5%)	27 (37.5%)	
**Gender**			0.006*
Female	78 (69.0%)	35 (31.0%)	
Male	72 (51.8%)	67 (48.2%)	
**Marital Status**			0.123
Married	61 (53.5%)	53 (46.5%)	
Other (single or divorced)	95 (62.9%)	56 (37.1%)	
**Family member in dentistry**			0.365
Yes	43 (64.2%)	24 (35.8%)	
No	107 (57.8%)	78 (42.2%)	
**Anticipated educational debt**			0.018*
< $100,000	22 (44.0%)	28 (56.0%)	
≥ $100,000	134 (62.3%)	81 (37.7%)	

*Significant at p < 0.05.

†Other plans include advanced dental education, military service, academics, etc.

Students planning to enter practice did not differ significantly from those with other career plans on the basis of marital status or having a family member in dentistry (Table 2). However, respondents who planned to enter practice immediately rated the influence of a family dentist, family other than spouse, and spouse’s occupation significantly higher than students with other career plans (Figure 1). Students who reported expected debt of at least $100,000 rated the influence of this debt on their career plans significantly higher than students with lower levels of debt (mean rating = 6.9 versus 4.8, respectively; p < 0.001).

Fifty-four percent of unmarried students rated the level of influence from their spouse as greater than 1, probably indicating that a significant other had some level of influence on career plans. Despite this discrepancy between reported marital status and spousal preferences (i.e., unmarried students who reported a high level of influence from spouses), married students rated this factor significantly more influential than unmarried students (mean level = 8.0 versus 3.5, respectively; p < 0.0001). A similar response pattern was seen between marital status and the influence of a spouse’s occupation; married students correspondingly rated the level of influence by this factor significantly higher than unmarried students (mean level = 4.6 versus 3.2, respectively; p = 0.001).

Dental students with an immediate family member who was a dentist or hygienist reported significantly greater mean influence on their career plans from their parent’s job than respondents without a family member in dentistry (mean level = 4.9 versus 1.8, respectively; p < 0.0001). Respondents with a family member in dentistry did not rate the influence of family members besides a spouse more highly than those without a family member in dentistry (p = 0.113).

Results of the logistic regression model are shown in Table 3. No evidence of multicollinearity among predictors was seen. The effects of gender on the likelihood of planning to enter practice immediately after graduation remained statistically significant in the multivariable model; female dental students were 1.95 times as likely as their male counterparts to plan to immediately enter practice. Marital status was not significantly associated with plans to enter practice in the bivariate analyses and it did not demonstrate significance in the multivariable model. However, an interaction between marital status and the influence of spouse’s occupation was significant in the regression model (p = 0.009).

**Table 3:** Multivariate logistic regression model for plans to enter private practice among fourth year dental students

	**Odds ratio (95% CI)**	**Sig.**
**Gender**		0.028*
Male	1	
Female	1.95 (1.08, 3.54)	
**Marital status**		0.242
Married	1.70 (0.70, 4.15)	
Other (single or divorced)	1	
**Anticipated educational debt**		0.023*
< $100,000	1	
≥ $100,000	2.34 (1.13, 4.87)	
**Family member in dentistry**		0.643
No	1	
Yes	1.18 (0.59, 2.35)	
**Influence of spouse’s occupation**^**†**^	1.25 (1.10, 1.43)	0.001*
**Influence of family other than spouse**^**†**^	1.08 (0.98, 1.18)	0.119
**Influence of family dentist**^**†**^	1.18 (1.06, 1.31)	0.002*
**Interaction:** [Marital status] × [Influence of spouse’s occupation]	0.78 (0.65, 0.94)	0.009*

^**†**^Influence rated from 1 (no influence) to 10 (very much).

*Significant at p ≤ 0.05.

For married dental students, there was no significant difference in the mean level of influence from a spouse’s occupation among those students who planned to enter private practice compared to students with other post-graduation career plans (4.5 versus 4.7, respectively; p = 0.73). However, among non-married individuals, students who planned to enter practice immediately reported a higher level of influence from a spouse’s occupation compared to students who had other career plans (3.9 versus 1.9, respectively; p < 0.0001).

Controlling for gender, marital status, and other variables in the full model, students with anticipated educational debt of $100,000 or greater were 2.34 times as likely as students with less anticipated debt to plan to enter practice immediately after graduation. Two other factors remained significantly related to the post-graduation career plans: spouse’s occupation and the influence of a family dentist. As the perceived influence from these two factors increased, the likelihood of planning to immediately enter private practice increased.

## Discussion

The logistic regression model demonstrated several factors to be significantly associated with dental students’ plans to enter private practice immediately after graduation: anticipated educational debt greater than $100,000, gender, the influence of a spouse’s occupation, and influence of a family dentist. No differences were found based on marital status or whether a dental student had an immediate family member in the field of dentistry.

There are several limitations to our study. This survey was administered to students from several graduating classes at only one institution, which may limit generalizability. Additionally, students were asked to rate how influential various factors rather than ranking these in order of influence, which may have resulted in different conclusions. Finally, asking students about future career plans may not accurately reflect what these students actually do after graduation. A follow-up survey of these new dentists within several years of graduation could lead to a better understanding of how different influences impact immediate career plans.

The cost of both public and private dental school continues to rise throughout the United States [2]. The 2013 projected tuition, fees, and expenses for an Iowa resident at the University of Iowa College of Dentistry is $190,000 for a four-year degree. For non-residents, projected costs are $281,000 [25]. This does not included cost of living expenses or the debt many students accrue during their four year bachelor’s degree, a prerequisite for almost all American dental schools.

Graduating dental students must decide whether to either enter the workforce immediately after graduation or to pursue another career option (e.g., advanced education or specialization). New dentists are leaving dental school with increasing levels of educational debt. While ADEA notes inconclusive evidence regarding the effect of educational debt on post-graduation career plans, our results show that increased debt was associated with increased likelihood to plan to enter general practice immediately after graduation. From 2000 through 2009, the average in-state tuition and fees for dental school in the U.S rose over 96% [26]. Recent ADEA data demonstrated that 41% of students rated their high levels of debt as having a substantial influence on their activity of choice after graduation [2].

Our study found that fourth year dental students with educational debt of at least $100,000 were more likely to plan to enter practice immediately after dental school than those with lower debt, even after adjusting for the influence of a spouse and others individuals who were identified as significantly influential on students’ career plans. Additionally, educational debt was the most highly influential factor regarding University of Iowa dental students’ plans after graduation. This concurs with reported findings that students with the greatest amount of anticipated debt planned on entering general practice or advanced education in general dentistry immediately after dental school [5].

Educational debt may influence new dentists to immediately enter the workforce in order to quickly reduce that burden. With less debt, students may otherwise choose to continue their education or may choose a different opportunity within dentistry. This effect may be especially pronounced in students from disadvantaged backgrounds who graduate with higher levels of debt. However, analysis of the 2007 ADEA survey found that non-underrepresented minorities were the most likely to graduate with the highest levels of debt nationally and that students with the highest levels of debt were three times less likely as students with less debt to enter public service [8].

Our results show female dental students plan on entering private practice immediately after graduation at a higher rate than their male counterparts; 69% of graduating females anticipated immediately entering practice, compared to 52% of males. Our finding is contrary to the trend from the 2011 ADEA survey of dental school seniors which found an approximately equal proportion of males and females immediately entered practice (48.7% and 49.%, respectively) [2].

Due to the increased role that women traditionally assume with child-rearing, their immediate career plans may reflect these responsibilities. Male dentists are more likely to be the principal earners in their family compared with females (85% of males versus 25% of females) [19]. Previous studies have also found that more females plan to pursue advanced education in general dentistry or pediatric dentistry than males; females that do pursue general practice residencies (GPR) or advanced education in general dentistry (AEGD) programs are then more likely to choose career paths in government, hospital care, or dental education rather than private practice [5,27].

Other influences were found to be significant factors predicting the likelihood of planning to enter practice immediately after dental school. The influence of a spouse’s occupation was significantly higher among those planning to immediately enter practice. Dental students who plan to immediately enter practice may have more pressure to hasten entry into the work force and generate income due to their spouses’ occupation, or lack thereof. Additionally, spouses may temporarily suspend their own career goals until dental students have graduated.

The influence of family members other than spouses and the influence of a family dentist were also rated significantly higher among those students planning to immediately entering practice. Positive family encouragement is important to the progression of students’ academic careers. Other studies have shown that strong encouragement from other influential parties, including spouses, relatives, mentors, and advisors can greatly increase pursuit of a specialty program [24].

The University of Iowa College of Dentistry is the only dental school in the state of Iowa and grants approximately 80 dental degrees each year - 60 of which are to state residents. The college offers all nine ADA-accredited specialty programs along with a general practice residency. Further research at the state and national levels should examine whether these findings have wider generalizability.

As the cost of receiving a dental education continues to rise, more students may choose to enter general dentistry immediately after graduation. However, longitudinal studies are needed to monitor whether this relationship remains the same over time. It is possible that once average debt reaches a certain point, students may decide that pursuing a dental specialty is the only feasible way to earn a high enough income to pay this down quickly.

## Conclusions

Fourth year students at the University of Iowa rated anticipated educational debt to be the most influential factor affecting their immediate career plans after dental school. Our model suggests that those students with debt at least $100,000, female students, those more influenced by their spouse’s occupation and family dentist are more likely to enter immediately into private practice. Our finding that women were more likely to anticipate entering practice immediately after dental school than males is counter to the national norm, where approximately equal numbers of men and women enter practice immediately after dental school [2]. As shown in this study, diverse influences affect the career plans of graduating dental students, but the importance of educational debt remained significant even after adjusting for these other influences.

## Competing interests

The authors declare that they have no competing interests.

## Authors’ contributions

BN, SM, FQ, and RK designed the study. SM performed data analysis. BN drafted the original paper. BN, SM, and RK provided comments on the original draft and contributed to the development of the final draft. All authors read and approved the final manuscript.

## Pre-publication history

The pre-publication history for this paper can be accessed here:

http://www.biomedcentral.com/1472-6831/14/18/prepub
